# Supplementary material for: Perovskite Chromite With In-Situ Assembled Ni-Co Nano-Alloys: A Potential Bifunctional Electrode Catalyst for Solid Oxide Cells
Source: Front Chem. 2021 Feb 1;8:595608. doi: 10.3389/fchem.2020.595608 (PMC7882693; doi:10.3389/fchem.2020.595608)
Supplement: Supplementary file 1 [file datasheet1.pdf]

Perovskite Chromite with *In-Situ* assembled Ni-Co Nano-Alloys: A Potential Bifunctional Electrode Catalyst for Solid Oxide Cells

Zhishan Li[1,a], Lin Cui[2,a], Jingli Luo[2], Jianhui Li[3], Yifei Sun[1,*]

1. College of Energy, Xiamen University, Xiamen, PR China

2. Department of Chemical and Materials Engineering, University of Alberta, Edmonton Canada

3. National Engineering Laboratory for Green Chemical Productions of Alcohols, Ethers and Esters, College of Chemistry and Chemical Engineering, Xiamen University, Xiamen, PR China

Abstract

Solid oxide fuel cell (SOFC) is an advanced electricity generation device with attractive fuel flexibility and conversion efficiency. As its reversed process, solid oxide electrolysis cell (SOEC) can efficiently electrolyze notorious CO_2 to valuable chemical product such as CO, by utilizing renewable energy. To achieve long-term operation, the development of catalytically active electrode materials in both SOFC/SOEC modes is highly desirable, yet still challenging. In this research, an A-site deficient perovskite oxide (lanthanum chromite) decorated with *in-situ* exsolved Ni-Co nano-alloy has been fabricated and applied as a potential fuel electrode for both SOFC/SOEC. The influences of A-site non-stoichiometry and B-site dopant concentration on structural properties and *in-situ* exsolution process have been elaborately studied from various aspects. Diverse characterizations collectively confirm that the existence of A-site deficiency helps the formation of oxygen vacancies and stimulates the exsolution of B-site cations. In addition, the synergistic effect between the dopants of Co and Ni manipulates the reducibility and promotes carbon deposition resistance of the material. The electrolyte-supported SOFC with self-assembled Ni-Co nano-alloy electrode has shown maximum power densities of 329 mW/cm^2 (in H_2) and 258 mW/cm^2 (in syngas, H_2+CO) at 850℃, which are 50% better than those of the fuel cell with the exsolved Ni nanoparticles only. Also, the nano-alloy decorated electrode catalyst promotes a 30% increase in SOEC performance for CO_2 electrolysis with prominently enhanced resistance against carbon deposition, suggesting the versatile functionality of the materials.

* Correspondence: Yifei Sun, yfsun@xmu.edu.cn

a. These authors contributes equally to this work.

Introduction

The rapid growth of the world's fossil fuel consumption in recent years has led to some severe environmental concerns such as greenhouse effect.[1] Therefore, many advanced technologies have been developed by researchers to alleviate these challenges. Fuel cell (FC) is a device that directly converts chemical energy to electricity beyond the limitation of Carnot cycle.[2] This process is also done in an eco-friendly manner with significant environmental advantages in terms of its low pollutant emission.

Working in the temperature range from 500 to 800 ˚C, the solid oxide fuel cell (SOFC) has been considered as the most efficient fuel cell with flexible fuel option including hydrocarbon fuels.[3] Additionally, the high operation temperature accelerates the kinetics of fuel oxidation, thus avoiding the use of noble metal catalysts and significantly decreasing the overall cost of system. Unfortunately, the current technology for SOFC is still difficult to achieve large scale commercialization mainly due to the unsatisfied fuel electrode (anode) performance and stability.[4] Fuel electrodes usually require high catalytic activities, good conductivities, high porosities, and compatibilities with electrolytes as well as interconnectors. Previously, the composite of Ni-YSZ cermet has been widely investigated as an anode for SOFC, which provides excellent ionic and electronic conductivities. However, its poor redox stability and rapid deactivation owing to carbon deposition still hinder its applications.[5]

An alternative candidate to substitute Ni-YSZ is perovskite oxide family which has an excellent stability at high temperature, wide structural tunability, as well as good compatibility with the commercial electrolyte material.[6] As a representative, the lanthanum chromite perovskite oxide, $LaCrO_{3-\delta}$, has an excellent chemical, mechanical and thermodynamic stability. The doping of the bivalent element including Sr or Ca at the A site leads to Cr^{3+}-to-Cr^{4+} transition, which creates more electronic holes in the valence band and thus improves the electronic conductivity. Meanwhile, the as-formed oxygen vacancies give rise to ionic conductivity by charge compensation (i.e., maintain electrical neutrality of the system).[7]

However, the application of lanthanum chromite in SOFC is still hindered by its low catalytic activity, mainly due to lack of reactive sites. To solve this problem, catalytically active metal nanoparticles were incorporated onto perovskite surface by wet impregnation (infiltration) or chemical deposition.[8] However, the agglomeration of the nanoparticles will inevitably occur after long-term operation due to the weak metal-oxide adhesion, resulting in the irreversible cell degradation.[9]

Alternatively, the *in-situ* exsolution method has been proposed to overcome this barrier in catalyst fabrication. The main conception of *in-situ* exsolution is to dissolve catalytic transition metals into the perovskite structure (at B sites) during the air preparation process (an oxidizing atmosphere), and force them to partially exsolve out of the lattice in a reducing atmosphere. Such process introduces the array of highly uniform dispersed nanoparticles on the perovskite support. Compared to infiltration method, the nanoparticles produced by *in-situ* exsolution were socketed into perovskite matrix, illustrating a stable metal particle/oxide support interface.[10] Also, according to the study by Neagu et al, the *in-situ* exsolved particles are expected to

maintain higher redox stability and better carbon deposition resistance while operating in hydrocarbon fuels.[11]

Besides fabrication methodology, introducing a second metal (guest metal) to construct a nano-alloy active site is expected to effectively manipulate the properties of metal/oxide catalyst. For example, Ni based alloys were confirmed to better suppress the formation of carbon fibers than pure Ni.[12] Takanabe et al. demonstrated that the catalytic activity of Ni-Co alloy catalyst gradually increases with the increased Ni content, while the coke resistance rises in proportion to the amount of Co content.[13] Grgicak et al. studied Ni-Co alloy in comparison to single Ni metal and pointed out that the Ni-Co alloy exhibited a highly stable activity as well as promoted the electrochemical activity in carbon containing environment over a wide range of temperature (500-900 °C).[14] Therefore, a proper ratio of Ni and Co needs to be considered for the tradeoff between reaction activity and coking resistance.

Based on the above consideration, in this work, a Ni-Co alloy assembled lanthanum chromite perovskite (LSC-NiCo) was prepared and the influences of Ni/Co content and cation deficiency on structural and chemical properties were systematically studied. The electrochemical performance of the electrode with exsolved Ni-Co alloy was evaluated in both SOFC and SOEC modes. Results show that the LSC-NiCo can be a promising candidate for reversible solid oxide cells.

Experiment Methodology

Synthesis of Cell Materials

Experimental Procedure of Electrode Fabrication

A citric acid & ethylene diamine tetra acetic acid (EDTA) complexing combustion method was applied to prepare the electrode materials. The precursor solution is formed by dissolving stoichiometric amounts of metal nitrates in deionized water with additive of citric acid ($C_6H_8O_7$) and EDTA ($C_{10}H_{16}N_2O_8$) as the co-chelating agents. Metal nitrates include lanthanum (III) nitrate hexahydrate ($La(NO_3)_3 \cdot 6H_2O$), strontium nitrate anhydrous ($Sr(NO_3)_2$), chromium (III) nitrate nona hydrate ($Cr(NO_3)_3 \cdot 9H_2O$), nickel (II) nitrate hexahydrate ($Ni(NO_3)_2 \cdot 6H_2O$), and cobalt (II) nitrate hexahydrate ($Co(NO_3)_2 \cdot 6H_2O$). The molar ratio of metal nitrate, EDTA and citric acid is 1:1:1.5. Then, the pH of solution is adjusted to ~8 using ammonium hydroxide (NH_4OH) for a better chelating result. The solution is subsequently stirred and heated at 80 ºC until the gel is formed. The gel is heated to 300 ºC rapidly and kept at 300 ºC for 0.5-1 h so that an auto combustion process of the gel will take place to form precursor powder (as-prepared powder). The precursor powders are then grounded and sintered at 1200 ºC for 5 h to form the single phase of targeted perovskite oxide. The designation of the materials is shown in Table 1.

Table 1 Designation of Bimetallic Doping Ratio Experiment

Abbreviation	Composition
LSC-6315NiCo	$(La_{0.6}Sr_{0.3})(Cr_{0.85}NiCo(3:1)_{0.15})O_{3-\delta}$
LSC-6312NiCo	$(La_{0.6}Sr_{0.3})(Cr_{0.88}NiCo(3:1)_{0.12})O_{3-\delta}$
LSC-6309NiCo	$(La_{0.6}Sr_{0.3})(Cr_{0.91}NiCo(3:1)_{0.09})O_{3-\delta}$
LSC-6306NiCo	$(La_{0.6}Sr_{0.3})(Cr_{0.94}NiCo(3:1)_{0.06})O_{3-\delta}$
LSC-6303NiCo	$(La_{0.6}Sr_{0.3})(Cr_{0.97}NiCo(3:1)_{0.03})O_{3-\delta}$
LSC-6309Ni	$(La_{0.6}Sr_{0.3})(Cr_{0.91}Ni_{0.09})O_{3-\delta}$
LSC-7309NiCo	$(La_{0.7}Sr_{0.3})(Cr_{0.91}NiCo(3:1)_{0.09})O_{3-\delta}$

The cathode of LSCF ($(La_{0.60}Sr_{0.40})_{0.95}Co_{0.20}Fe_{0.80}O_{3-\delta}$) was purchased from Fuel Cell Materials used as the air electrode in this work.

Fabrication of Solid Oxide Cells

Electrode ink was fabricated by thoroughly mixing the electrode powder, the GDC10 power (10% gadolinium doped ceria oxide, Fuel Cell materials) and the electrode glue at the weight ratio of 1.5:1.5:1.7 in the milling

machine for 2 h. The GDC buffer layers introduced at the electrode and electrolyte interfaces were made by mixing the GDC10 powder with the electrode glue at 1.7:3 weight ratio. Buffer layers were painted on the two sides of the electrolyte (25 mm diameter, 0.3 mm thickness, 8 mol% yttrium doped zirconia oxide, Fuel Cell Materials). Buffer layers were dried in an air-drying oven (at 90 ºC for 15 min) and sintered with the electrolyte at 1300 ºC for 5 h. Then the fuel electrode ink was painted on one side of the buffer layer with the painting area of 0.965 cm^2. After air-drying the ink, the fuel electrode LSC was sintered at 1200 ºC for 5 h. The air electrode LSCF was painted on the other side of the cell, then sintered at 950 ºC for 4 h in the furnace to obtain good adhesion between the electrode and electrolyte. Au paste was painted on both sides of the electrode as the current collector.

Electrochemical Test Setup for SOFC

The well fabricated cells were placed in an electrochemical test setup shown in Figure S1. The fabricated cell was sealed on a home-made coaxial alumina two-tube setup by a ceramic sealant (Ceramabond 552, Aremco Products) to separate air and fuel gas injected into the different chambers. The current collectors for the two electrodes were connected to the electrochemical workstation (potentiostat) by 0.5 mm diameter silver wires. The volumetric flow meters were used to control the flow rate of inlet gases, and the outlet gases flowing at the cathode and anode were removed from the system to carry away the generated products and thus allow the reaction to continue. Once the ceramic sealant was cured at room temperature in air, this set-up would be placed in the Thermolyne tubular furnace and then heated to 70 and 260 ºC for 1 h with a ramping rate of 1 ºC min^{-1}. Finally, it was heated to the operating temperature with a ramping rate of 2 ºC min^{-1} for testing. The electrochemical performance was evaluated using the electrochemical workstation with a Solartron 1287 potentiostat and a Solartron 1255 frequency response analyzer to collect data and measure the produced powers from SOFC operations (Figure S1).

Material Characterizations

The phase of the catalyst was analyzed by powder X-Ray diffraction (XRD) using a Rigaka D/max-2500 X-ray diffractometer with a Cu Kα radiation at room temperature and the data were analyzed with Jade and Xpert Highscore Plus Software. The microstructure and morphology of the catalysts, cross sectional and surface images of the cells as well as the material composition by SEM-EDS analysis were obtained at room temperature by Zeiss Sigma 300VP-FESEM equipment. Thermogravimetric (TGA) Analysis of the materials was performed using a Q600 (TA instrument) instrument in different atmospheres. The temperature program oxidation (TPO) was carried out using a TG-Mass Spectrometry (thermostat QMS 200) instruments (TG-MS). The hydrogen-temperature program reduction (H_2-TPR) analysis was performed using a home-made temperature program setup equipped with a Hewlett Packard 5890 Series II gas chromatograph. The X-ray photoelectron spectra were collected on a Thermo fisher Scientific K-$Alpha^+$ instrument. The C1s XPS peak was calibrated to 284.6 eV, as shown in Figure S2.

Results

The powder X-ray diffraction (XRD) was used to confirm the crystalline structures of the fabricated materials. According to the study on the materials with different Co/Ni percentages, the optimal concentration of B-site dopant was confirmed as 9 mol% (Figure S3), and higher doping content will lead to co-existence of NiO impurity. Furthermore, we manipulated the compositions of the material by varying the A-site deficiency and Ni/Co ratio. Figure 1a and 1b shows the corresponding XRD patterns. The three materials, LSC-6309NiCo, LSC-6309Ni and LSC-7309NiCo all successfully formed the single phase of LSC perovskite oxide as marked by the asterisk (*).[15] Moreover, some minor peak shift could be identified by analyzing peak position in detail (Figure 1b). The (104) diffraction peaks for LSC-6309NiCo, LSC-6309Ni and LSC-7309NiCo were at 32.75°, 32.85°, and 32.65° (2θ), respectively. According to Bragg Law ($n\lambda = 2d \sin\theta$), the LSC-6309Ni should have the smallest d-spacing and cell parameter while LSC-7309NiCo has the largest unit cell. The evolution of cell volume can be preliminarily explained by the different ion radius of dopants. The ionic radii for 6-fold coordination B-site Cr^{3+}, Ni^{2+} and Co^{2+} are 0.615, 0.69 and 0.745 Å respectively.[16] Thus, it is reasonable to expect that the bimetallic Ni-Co doping has the increased unit cell volume. Also, the decrease of the unit cell volume of LSC-6309NiCo compared with LSC-7309NiCo is mainly caused by the A-site cation non-stoichiometry, which is in agreement with the literature results of A. V. Kovalevsky et al.[17]

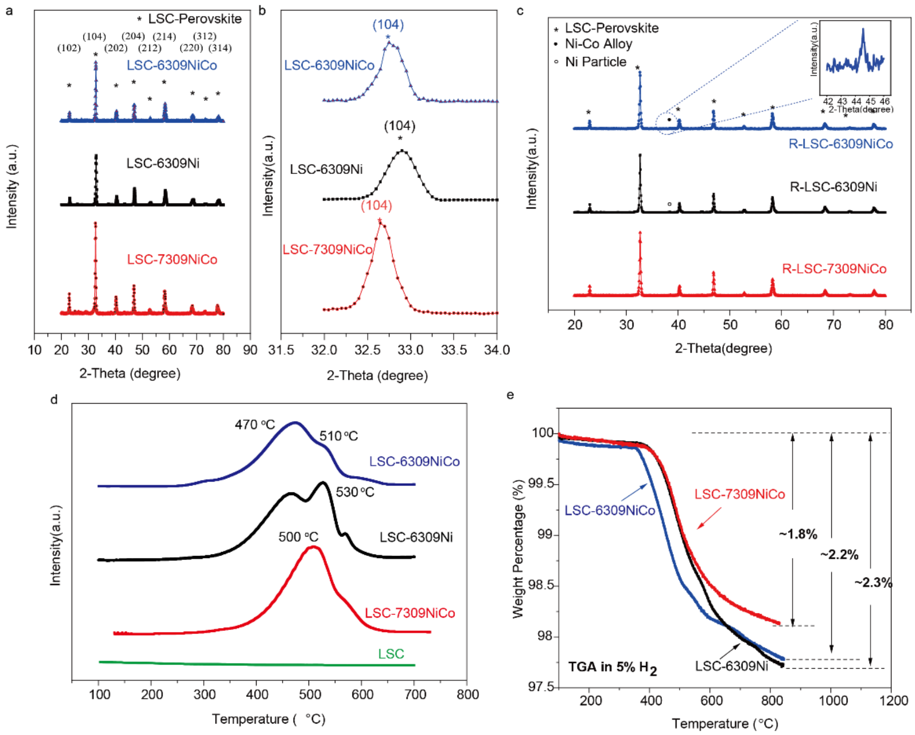

Figure 1. a) XRD patterns and b) the partial enlarged detail for perovskite oxide LSC-6309NiCo, LSC-6309Ni and LSC-7309NiCo, c) XRD patterns for the reduced samples R-LSC-6309NiCo, R-LSC-6309Ni and R-LSC-7309NiCo, d) H_2-TPR curves of the fresh LSC, LSC-6309NiCo, LSC-6309Ni and LSC-7309NiCo, and e) TGA diagram in 5%H_2/N_2 for LSC-6309NiCo, LSC-6309Ni and LSC-7309NiCo.

With *in-situ* exsolution treatment on various samples, the metallic particles are expected to be small in size (nanoscale) and uniformly decorated on the surface of the LSC parent. The tendency of the exsolution of metal particles can be partially predicted by the Gibbs free energy. The values of Gibbs free energy of the reduction reaction of each cation were calculated using HSC 6.0 software and shown in Figure S4. Only Co and Ni are thermodynamically favorable to exsolve under the high temperature from 600 to 900 ℃, since only the reductions of Co_3O_4 and NiO have the negative Gibbs free energies. For example, at 800 ℃, the reduction of Co_3O_4 to Co has a Gibbs free energy of -256.91 kJ/mol and that of NiO to Ni is -46.646 kJ/mol, while all other elements still show positive values. In addition, the exsolved Ni and Co metallic particles are expected to form a Ni-Co alloy phase during the high temperature *in-situ* exsolution treatment on the surface of perovskite. Theoretically, it is known that Co and Ni are adjacent to each other in the periodic table and satisfy the Hume-Rothery rule, indicating that they can easily form a solid solution phase (alloy) with various ratios.[18] Thus, it is tentatively suggested that the formation of Ni-Co alloy during the exsolution process in this experiment is favorable.

Based on these theoretical estimations, all the samples were reduced at 800 ℃ for 3 h in 5% H_2/N_2 atmosphere during which the dopants (Ni, Co) should be *in-situ* exsolved out of the bulk LSC and get uniformly dispersed on the surface. The XRD patterns for reduced samples R-LSC-6309NiCo, R-LSC-6309Ni and R-LSC-7309NiCo, and their detailed exsolution peak positions are illustrated in Figure 1c. All of the reduced samples have successfully maintained the main LSC structure after the high temperature reduction process as indicated by asterisk-marked peaks. The diffraction peak labelled as hollow circle has been detected for LSC-6309Ni, indicative of the formation of metallic Ni. In addition, a clear diffraction peak labelled as solid circle has been detected for both R-LSC-6309NiCo and R-LSC-7309NiCo, which can be assigned to Ni-Co alloy.[19]

To investigate the reducibility of this series of materials and the synergistic effect between Co and Ni, hydrogen temperature programmed reduction (H_2-TPR) tests were carried out. As shown in Figure 1d, there is no obvious H_2 consumption peak occurring in the pattern of LSC perovskite, indicating that LSC might not have exsolved nanoparticles in a reducing atmosphere with increasing temperature. This result is consistent with the Gibbs free energy diagram as mentioned before. Doping of active metals Ni and Co has made a great improvement on the reducibility of the material. Clear H_2 consumption peaks are observed for both LSC-6309NiCo and LSC-6309Ni. The curve of LSC-6309Ni has two clear H_2 consumption peaks at ~470 °C and 530 °C. Based on the previous studies,[20] the first peak (α peak) of LSC-6309Ni corresponds to the reduction of Ni^{3+} to Ni^{2+} (around 500°C), while the second peak (β peak) represents the reduction from Ni^{2+} to metallic Ni^0 (before 600°C). The Ni^{2+} to Ni^0 transformation consumes a large amount of hydrogen, which can be confirmed from the area of the β peak because the usage of samples for H_2-TPR measurement is the same. The LSC-6309NiCo material has a similar shape of α peak at ~470 °C, but with a much lower β peak at 510 °C. The material with different B-site concentration shows similar shape of TPR plots (Figure S5). This phenomenon indicates that the formation of the Ni-Co solutions significantly changes the reduction behavior of the material. A previous study indicated that the main H_2-TPR peak for Co_3O_4 occurs at 433 °C,

which just overlapped with α peak of Ni.[21] That can also explain the wider range of α peak in LSC-6309NiCo than in LSC-6309Ni. The β peak for LSC-7309NiCo has moved to a lower temperature and partially merged with the α peak. This result means that the exsolution process of LSC-7309NiCo requires higher energy than LSC-6309NiCo for both Ni^{3+} to Ni^{2+} and Ni^{2+} to Ni^0 transformations. The TPR result clearly proves that the addition of the second metal Co can significantly facilitate the reduction of materials. The existence of A-site deficiency could also accelerate the reduction to generate more Ni-Co nanoparticles.

As the *in-situ* exsolution proceeds, the coordinated lattice oxygen ions surrounding the cations escape from the LSC perovskite oxide as well. The non-stoichiometric oxygen ions have introduced fair amount of oxygen vacancies (δ) into the structure. The quantity of oxygen vacancies plays a significant role in the ionic conductivity and electrochemical catalytic activity of a perovskite material. In this experiment, the TGA was performed to measure the weight losses of the materials with increasing temperature in the reducing atmosphere. As stated by previous studies, the primary weight loss in the curve is due to the formation of oxygen vacancies.[21-22] The TGA was operated for different materials from 100 to 850 °C at a heating rate of 20 °C per minute in 5% H_2/N_2. Figure 1e shows the comparison of TGA curves for LSC-6309NiCo, LSC-6309Ni, and LSC-7309NiCo, and the weight losses for them are ~2.2%, 2.3%, and 1.8%, respectively. LSC-6309NiCo had a similar weight loss to that of LSC-6309Ni, but had an obvious lower onset temperature and a larger weight loss in stage two and the first half of stage three (similar as LSC-6303NiCo and LSC-6306NiCo shown in Figure S6). Stages two and three are the main steps to introduce oxygen vacancies into the structure, and LSC-6309NiCo started at 380 and 510 °C while LSC-6309Ni at 420 and 580 °C. At the last part of stage three, at around 600 °C, the weight loss of LSC-6309Ni increased, resulting in a similar increased amount for LSC-6309NiCo. This result indicates that A-site deficient material, both from monometallic doping and bimetallic doping, shall introduce a similar amount of oxygen vacancies into the structure, but bimetallic doped perovskite demonstrates a facilitated exsolution process. In comparison, LSC-7309NiCo has a smaller amount of weight loss than both LSC-6309NiCo and LSC-6309Ni, and the starting temperatures for stage two and three are around 420 and 580 °C, which are higher than that for LSC-6309NiCo. By comparing the result of these TGA curves, it can be inferred that the introduction of A-site deficiency and bimetallic doping of Ni and Co can facilitate the formation of oxygen vacancies into the structure, thereby improving ionic conductivity and catalytic activity.

The identification for the exsolution is further analyzed by SEM microstructure images. Figure 2a shows the SEM image of fresh LSC-6309NiCo powder sample. Clearly, no exsolution of metallic nanoparticles could be found on the surface of fresh sample with the particle size of around 500 nm. In comparison, numerous amounts of exsolved nanoparticles are observed on the reduced sample R-LSC-6309NiCo, which was pre-treated in 5% H_2-N_2 at 800 °C for 4 h, as shown in Figure 2b. The exsolved particles are uniformly dispersed on the surface of bulk. Figures 2c and 2d are the point scanning results for R-LSC-6309NiCo. The bulk phase in Figure 2c is marked as point (1) and the exsolved particle is marked as point (2). From Figure 2d, La is detected at 0.5, 4.7, 5.1 and 5.9 keV, Sr at 1.8 keV, and Cr at 0.25 and 5.4 keV for both the bulk and exsolved

particle. Moreover, distinct Co and Ni peaks are detected at point (2) at 7 and 7.5 keV, respectively, while the peaks for point (1) are weak at these positions. Similar method was also applied to confirm the formation of Ni nanoparticle in R-LSC-6309Ni (see Figure S7). It is concluded that the exsolution particles from R-LSC-6309NiCo base are indeed Ni-Co alloy. The TEM images in Figure 3 further confirms the Ni-Co alloy structure of exsolved particle on the surface of R-LSC-6309NiCo. Herein, the exsolved particle is marked as point (3).

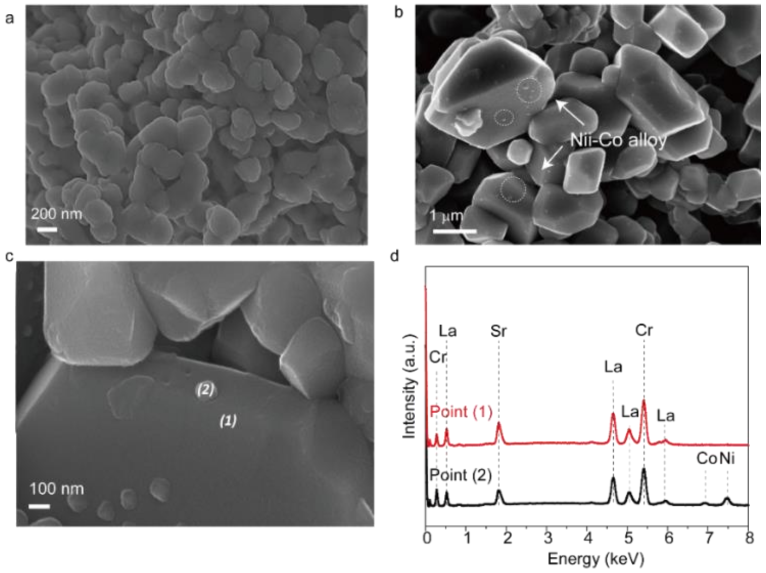

Figure 2. SEM images of a) fresh LSC-6309NiCo microstructure, b) reduced sample R-LSC-6309NiCo, and c)-d) the correlated EDS point scanning results.

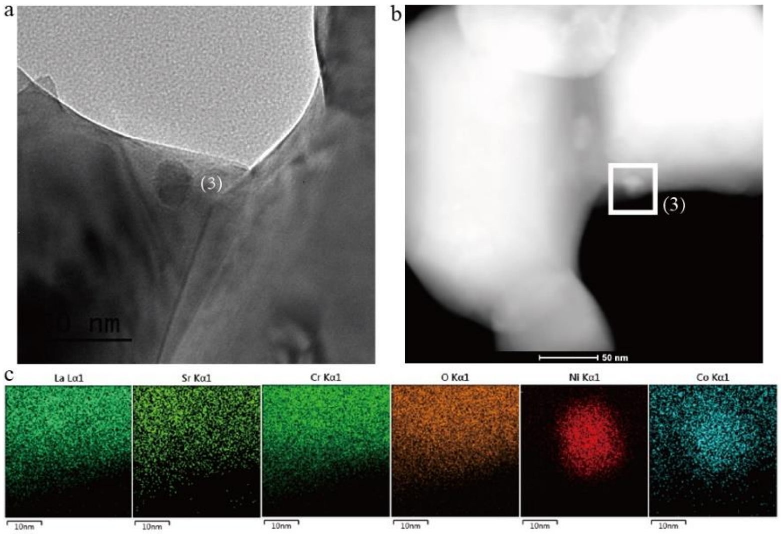

Figure 3. a) TEM and b) STEM-HAADF images of the reduced sample R-LSC-6309NiCo, and c) the correlated elemental mapping of La, Sr, Cr, O, Ni and Co for point (1) in b), which is represented by cyan, lime, green, orange, red and blue, respectively.

To understand the evolution of element composition and valence state of samples before and after the exsolution, the X-ray photoelectron spectroscopy spectra (XPS) were further employed. As shown in Figure 4a, the Ni2p XPS spectra are similar to that of NiO, suggesting its major composition of Ni^{2+}.[23] After exsolution treatment, the peak positions of Ni2p1/2 and Ni2p3/2 both shift to lower binding energy regime by 0.61 eV, which suggests a significant valence decrease of Ni after reduction and the formation of metallic state Ni.[24] The O1s XPS spectra of different samples are shown in the Figure 4b. The percentage of oxygen species obtained by XPS peak fitting is shown in Table S1. There are huge differences in the O1s XPS spectra of the samples before and after the exsolution. The high resolution O1s spectrum can be deconvoluted into four peaks at 532.8, 530.1, 530.3 and 528.8 eV, which can be ascribed to surface absorbed molecular oxygen species, surface adsorbed oxygen/hydroxyl group, surface adsorbed oxygen/hydroxyl group and lattice oxygen species, respectively. After exsolution, the R-LSC-6309NiCo represents enhanced concentration of O_2/OH (38.58%) and O_2^{2-}/O^- (21.50%), suggesting the higher content of oxygen vacancy.[25] The larger amount of oxygen vacancies on the samples may also contribute to the ionic diffusion and facilitate the catalytic reaction. Similar to Ni2p XPS spectra, the Co2p3/2 and Co2p1/2 XPS spectra of samples after reduction have a blue shift by 0.55 eV. The data collected from XPS are consistent with the characterization of XRD and SEM, confirming the formation of Ni-Co alloy.

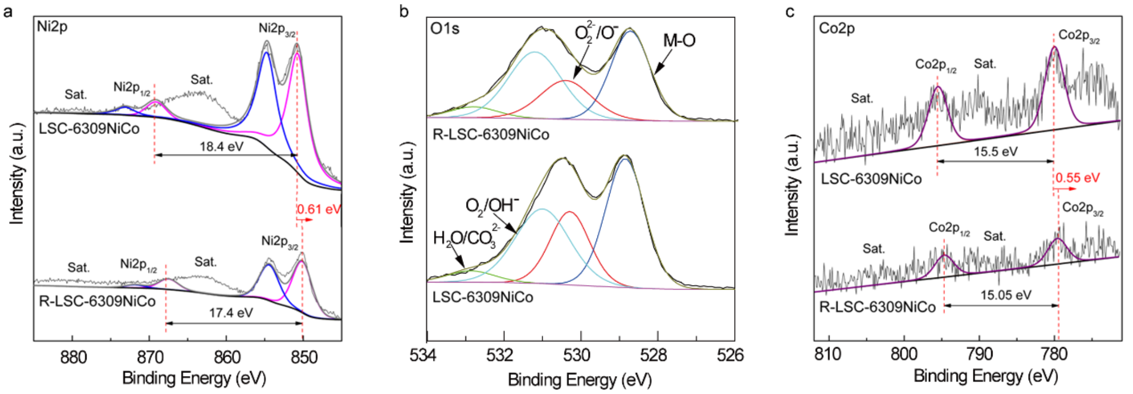

Figure 4. XPS spectra of LSC-6309NiCo and R-LSC-6309NiCo samples: a) Ni2p, b) O1s and c) Co2p.

The SEM image of the cross-section microstructure for the tested cell is shown in Figure 5a. The dense YSZ electrolyte with a thickness of 300 μm can be observed, and two 15 μm GDC buffer layers were introduced between the electrode and the electrolyte. The fuel and air electrode layers with the thickness of approximately 30 μm were applied above the GDC layers. Figure 5b shows the j-V curve for LSC-6309NiCo and LSC-6309Ni (anode) cells at 850°C in pure H_2 gas. The tested cells were reduced with 5% H_2/N_2 gas for 2 h before the test to trigger the *in-situ* exsolution of Ni-Co alloy. In H_2 gas, the OCV values for LSC-6309NiCo and LSC-6309Ni cells were 1.15 and 1.13 V, respectively. The high values of OCV observed indicated that negligible gas leakage was present in the cell. The LSC-6309NiCo cell can generate a maximum power density of 329 mW/cm^2 with a current density of 599 mA/cm^2, while the LSC-6309Ni cell

only had a maximum power output of 237 mW/cm^2 with a current density of 441 mA/cm^2. Figure 5c illustrates the electrochemical performance for LSC-6309NiCo and LSC-6309Ni cells in syngas at 850°C. The OCV values of 1.11 and 1.14 V were determined as expected for LSC-6309NiCo and LSC-6309Ni, respectively, in syngas. LSC-6309NiCo cell produced a maximum power density of 258 mW/cm^2 with current density of 455 mA/cm^2, as compared to the LSC-6309Ni cell having a maximum power density of 170 mW/cm^2 and with a current density of 307 mA/cm^2. These results demonstrate that the Ni-Co alloy doped LSC cell can maintain the promoted catalytic activity for both H_2 and syngas oxidations.

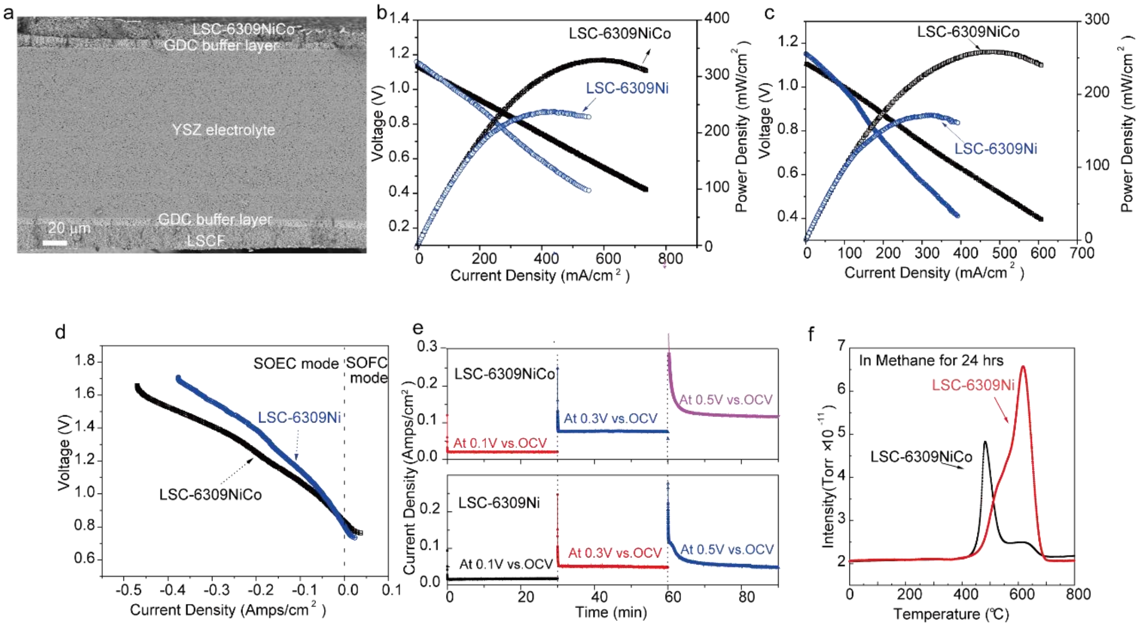

Figure 5. a) SEM image of the cross-section area of the cell, b) j-V curves of LSC-6309NiCo and LSC-6309Ni at 850 °C in pure H_2 gas and c) in syngas, d) j-V curves of LSC-6309NiCo and LSC-6309Ni at 850°C in 70% CO_2/ 30%CO, e) Short term stabilities of the electrolysis cells with LSC-6309NiCo and LSC-6309Ni cathodes at 850 °C at various applied voltages, and f) TPO curves for CH_4 treated LSC-6309NiCo and LSC-6309Ni.

Figure S8a illustrates the EIS performance of both LSC-6309NiCo and LSC-6309Ni cells from 10^6 to 0.1 Hz under open circuit voltage condition with H_2 gas at 850 °C. The curves intersections with the real axis (x-axis) at high frequencies represent their ohmic resistance, which is mainly caused by the electrolyte material.[26] LSC-6309NiCo and LSC-6309Ni cells have shown the similar ohmic resistances of 0.54 and 0.56 Ω, which are reasonable with the 300 μm thickness of YSZ electrolyte. The difference between the two intersections at high frequency and low frequency with the x-axis represents the activation polarization resistance, which is the sum of the electrode (anode and cathode) reaction resistances.[27] The real parts of the impedance at low frequency for LSC-6309NiCo and LSC-6309Ni are partially compared at 10, 1 and 0.1 Hz as marked by the dashed lines in the figure. LSC-6309NiCo cell has shown a clear reduction of the real impedance compared to LSC-6309Ni at the identical frequencies. This result has demonstrated the lower

polarization resistance of LSC-6309NiCo cell compared with the LSC-6309Ni cell. The lower value of polarization resistance for the LSC-6309NiCo also clarifies its better electrochemical performance. Figure S8b is the EIS diagram for LSC-6309NiCo and LSC-6309Ni cells for syngas oxidation at 850 °C under OCV condition. Compared with the H_2 oxidation EIS diagram, no significant increase of ohmic resistances are found in syngas atmosphere. In addition, the real impedance values of each cell are compared at 10, 1 and 0.1 Hz. Apparently, LSC-6309Ni has an obvious larger value of real impedance than LSC-6309NiCo under each frequency.

Furthermore, the electrochemical performances of SOEC using LSC-6309NiCo and LSC-6309Ni cathodes were evaluated at 850 °C in CO_2/CO atmosphere at the ratio of 70:30. CO gas was added into fuel gas CO_2 during the electrochemical test to prevent the oxidation of exsolved particles. The cells were tested mainly under electrolysis mode (negative current density) with applied voltages from 0V to 0.9V (vs. OCV). Figure 5d illustrates the j-V curve of SOEC equipped with LSC-6309Ni and LSC-6309NiCo cathodes. The OCV values for both cells are 0.84-0.82 V, which are close to the theoretical values in 70% CO_2/ 30% CO atmosphere. As depicted, the LSC-6309NiCo cell has current densities of 0.039, 0.136 and 0.245 mA/cm^2 at the applied voltages of 0.1, 0.3 and 0.5 V, respectively, while LSC-6309Ni has corresponding current densities of 0.027, 0.089 and 0.179 mA/cm^2. The LSC-6309NiCo cell has expressed a better performance than the LSC-6309Ni cell in CO_2/CO atmosphere at all applied voltages, which demonstrates that the bimetal doped LSC cell has a better catalytic property in the CO_2 reduction process.

Short term operating stabilities for LSC-6309NiCo and LSC-6309Ni electrolysis cells were tested in 70% CO_2/30% CO at 850 ℃ with various applied voltages and plotted in Figure 5e. Both cells were run in the SOEC mode for 30 min at constant voltages of 0.1, 0.3 and 0.5 V (vs. OCV). The LSC-6309NiCo electrolysis cell shows good stabilities under all voltages and the current densities of the cell with increasing applied voltage. In contrast, the LSC-6309Ni electrolysis cell expresses good stabilities at the applied voltages of 0.1 and 0.3 V. A significant degradation of the LSC-6309Ni cell was detected at 0.5V applied voltage. The possible reason for this degradation might be the influence from carbon deposition on Ni particles from the over reduction in CO_2/CO atmosphere.[28] As a result, the stable operating potential for LSC-6309Ni can be inferred as 0.3 V vs. OCV. The morphology of bulk LSC materials is maintained after short-term tests, as presented in Figure S9. Overall, the bimetallic doped LSC perovskite has shown better stability than the Ni doped LSC in an SOEC atmosphere at various applied potentials.

Previous studies have indicated the poor carbon deposition resistance of Ni particle doped fuel electrodes operating in coal gases. The coal derived fuel gas applied to solid oxide cells such as hydrocarbons, syngas, and even CO_2 gas can cause carbon deposition and lead to performance degradation of the cell.[29] Therefore, carbon deposition resistance is another important parameter for catalytic activity analysis of the electrode. The carbon deposition resistance of Ni and Ni-Co LSC perovskite oxide was studied in this work by O_2-TPO analysis. The samples were sintered with CH_4 gas at 850 ℃ for 24 h. After the treatment with CH_4 gas, the samples were inserted for the TGA test from 0℃ to 850 ℃ in air. The gas outlet of the TGA was connected

with the MS, therefore, the specific amount of produced CO_2 at different temperatures could be measured. The O_2-TPO profile measured by MS is shown in Figure 5f. The CO_2 peak areas for LSC-6309NiCo and LSC-6309Ni were 4.21×10^{-9} and 5.30×10^{-9}, respectively. From this result, it can be concluded that compared with Ni doped catalytic material, the addition of Co into the catalyst can effectively suppress the formation of carbon deposition on the electrode material as studied by K. Takanabe et al.[13] Moreover, the CO_2 peak position of LSC-6309Ni electrode material is at around 620 °C, while the peak position for LSC-6309NiCo is around 470 °C. The lower operating temperature for LSC-6309NiCo electrode material indicates a smaller energy required for redox treatment. Compared to LSC-6309Ni, LSC-6309NiCo shows higher carbon deposition resistance and lower required recovery temperature. This is because the formation of surface Ni-Co alloy can effectively suppress the bonding of undesired carbon and preferentially oxidize the carbon atom into gas phase. The monometallic Ni is more likely to facilitate the formation of carbon-carbon bonds,[30] resulting in poor carbon deposition resistance of LSC-6309Ni.

Conclusions

The LSC-based perovskite oxides were prepared in this work as a potential electrode material for SOFC and SOEC. The roles of A-site stoichiometry and B-site dopant concentration on material properties were studied by XRD, TPR, TPO, TGA, XPS and SEM. LSC-6309NiCo and LSC-6309Ni were selected for the electrochemical performance tests in SOFC and SOEC. The following conclusions can be drawn based on the experimental results:

- The Ni and Co doped LSC perovskite oxide were fabricated by combustion process with the saturation doping level up to 9 mol%.
- The introduction of A-site deficiency is the key driving force to trigger the exsolution of Nano Ni-Co alloy particles. The exsolution process was found to be facilitated by Co incorporation.
- The Ni-Co alloy decorated materials delivered better electrochemical performance in both SOFC and SOEC modes. Meanwhile, the formation of Ni-Co alloy has efficiently reduced the carbon deposition in the cell while operating in SOEC mode.

Acknowledgements

Y. Sun acknowledges the support from the Nanqiang Young Top-notch Talent Fellowship from Xiamen University. This work is supported by the National Natural Science Foundation of China (No.21773195). This research is financially supported by the Natural Sciences and Engineering Research Council of Canada-Discovery Grant (GRPIN–2016–05494), and Alberta Innovates Technology Futures Research Grant. As a part of the University of Alberta's Future Energy Systems research initiative, this research was made possible in part thanks to funding from the Canada First Research Excellence Fund.

References

1. Pérez-Lombard, L.; Ortiz, J.; Pout, C., A review on buildings energy consumption information. *Energy and buildings* **2008,** *40* (3), 394-398.
2. O'hayre, R.; Cha, S.-W.; Colella, W.; Prinz, F. B., *Fuel cell fundamentals*. John Wiley & Sons: 2016.
3. Stambouli, A. B.; Traversa, E., Solid oxide fuel cells (SOFCs): a review of an environmentally clean and efficient source of energy. *Renewable and sustainable energy reviews* **2002,** *6* (5), 433-455.
4. Da Silva, F. S.; de Souza, T. M., Novel materials for solid oxide fuel cell technologies: A literature review. *international journal of hydrogen energy* **2017,** *42* (41), 26020-26036.
5. Prakash, B. S.; Kumar, S. S.; Aruna, S., Properties and development of Ni/YSZ as an anode material in solid oxide fuel cell: a review. *Renewable and Sustainable Energy Reviews* **2014,** *36*, 149-179.
6. Sunarso, J.; Hashim, S. S.; Zhu, N.; Zhou, W., Perovskite oxides applications in high temperature oxygen separation, solid oxide fuel cell and membrane reactor: A review. *Progress in Energy and Combustion Science* **2017,** *61*, 57-77.
7. (a) Sarno, C.; Luisetto, I.; Zurlo, F.; Licoccia, S.; Di Bartolomeo, E., Lanthanum chromite based composite anodes for dry reforming of methane. *International Journal of Hydrogen Energy* **2018,** *43* (31), 14742-14750; (b) Setz, L.; Santacruz, I.; León-Reina, L.; De la Torre, A.; Aranda, M.; Mello-Castanho, S.; Moreno, R.; Colomer, M., Strontium and cobalt doped-lanthanum chromite: Characterisation of synthesised powders and sintered materials. *Ceramics International* **2015,** *41* (1), 1177-1187.
8. Sfeir, J.; Buffat, P. A.; Möckli, P.; Xanthopoulos, N.; Vasquez, R.; Mathieu, H. J.; Thampi, K. R., Lanthanum chromite based catalysts for oxidation of methane directly on SOFC anodes. *Journal of Catalysis* **2001,** *202* (2), 229-244.
9. Jiang, S. P., A review of wet impregnation—an alternative method for the fabrication of high performance and nano-structured electrodes of solid oxide fuel cells. *Materials Science & Engineering A* **2006,** *418* (1-2), 199-210.
10. Kwon, O.; Joo, S.; Choi, S.; Sengodan, S.; Kim, G., Review on exsolution and its driving forces in perovskites. *Journal of Physics: Energy* **2020**.
11. Neagu, D.; Oh, T.-S.; Miller, D. N.; Ménard, H.; Bukhari, S. M.; Gamble, S. R.; Gorte, R. J.; Vohs, J. M.; Irvine, J. T., Nano-socketed nickel particles with enhanced coking resistance grown in situ by redox exsolution. *Nature communications* **2015,** *6* (1), 1-8.

12. An, W.; Gatewood, D.; Dunlap, B.; Turner, C. H., Catalytic activity of bimetallic nickel alloys for solid-oxide fuel cell anode reactions from density-functional theory. *Journal of Power Sources* **2011,** *196* (10), 4724-4728.
13. Takanabe, K.; Nagaoka, K.; Aika, K.-i., Improved resistance against coke deposition of titania supported cobalt and nickel bimetallic catalysts for carbon dioxide reforming of methane. *Catalysis Letters* **2005,** *102* (3-4), 153-157.
14. Grgicak, C. M.; Pakulska, M. M.; O'Brien, J. S.; Giorgi, J. B., Synergistic effects of Ni1− xCox-YSZ and Ni1− xCux-YSZ alloyed cermet SOFC anodes for oxidation of hydrogen and methane fuels containing H2S. *Journal of Power Sources* **2008,** *183* (1), 26-33.
15. Pudmich, G.; Boukamp, B. A.; Gonzalez-Cuenca, M.; Jungen, W.; Zipprich, W.; Tietz, F., Chromite/titanate based perovskites for application as anodes in solid oxide fuel cells. *Solid State Ionics* **2000,** *135* (1-4), 433-438.
16. Sebastian, M. T., *Dielectric materials for wireless communication*. Elsevier: 2010.
17. Kovalevsky, A.; Yaremchenko, A.; Populoh, S.; Weidenkaff, A.; Frade, J., Effect of A-site cation deficiency on the thermoelectric performance of donor-substituted strontium titanate. *The Journal of Physical Chemistry C* **2014,** *118* (9), 4596-4606.
18. Janghorban, K.; Kirkaldy, J.; Weatherly, G., The Hume-Rothery size rule and double-well microstructures in gold-nickel. *Journal of Physics: Condensed Matter* **2001,** *13* (38), 8661.
19. Kwon, O.; Kim, K.; Joo, S.; Jeong, H. Y.; Shin, J.; Han, J. W.; Sengodan, S.; Kim, G., Self-assembled alloy nanoparticles in a layered double perovskite as a fuel oxidation catalyst for solid oxide fuel cells. *Journal of Materials Chemistry A* **2018,** *6* (33), 15947-15953.
20. (a) Jahangiri, A.; Aghabozorg, H.; Pahlavanzadeh, H., Effects of Fe substitutions by Ni in La–Ni–O perovskite-type oxides in reforming of methane with CO2 and O2. *International journal of hydrogen energy* **2013,** *38* (25), 10407-10416; (b) Rida, K.; Peña, M.; Sastre, E.; Martinez-Arias, A., Effect of calcination temperature on structural properties and catalytic activity in oxidation reactions of LaNiO3 perovskite prepared by Pechini method. *Journal of Rare Earths* **2012,** *30* (3), 210-216.
21. Konsolakis, M.; Sgourakis, M.; Carabineiro, S. A., Surface and redox properties of cobalt–ceria binary oxides: on the effect of Co content and pretreatment conditions. *Applied Surface Science* **2015,** *341*, 48-54.
22. He, W.; Huang, H.; Chen, M.; Gao, J.-F.; Chen, C.-S., Stability and oxygen transport property of La0. 8Sr0. 2Cr0. 5Fe0. 5O3-δ. *Solid State Ionics* **2014,** *260*, 86-89.

23. Du, X.; Ding, Y.; Li, C., Morphology-Controlled Self-Assembly and Nanostructured NiO: An Efficient and Robust Photocatalytic Water-Oxidation Catalyst. *ChemCatChem* **2015,** *7* (15), 2370-2376.
24. Liu, Y.; Fu, N.; Zhang, G.; Lu, W.; Zhou, L.; Huang, H., Ni@ NiO core/shell dendrites for ultra-long cycle life electrochemical energy storage. *Journal of materials chemistry A* **2016,** *4* (39), 15049-15056.
25. Zhu, Y.; Zhou, W.; Yu, J.; Chen, Y.; Liu, M.; Shao, Z., Enhancing electrocatalytic activity of perovskite oxides by tuning cation deficiency for oxygen reduction and evolution reactions. *Chemistry of Materials* **2016,** *28* (6), 1691-1697.
26. Fabbri, E.; Pergolesi, D.; D'Epifanio, A.; Di Bartolomeo, E.; Balestrino, G.; Licoccia, S.; Traversa, E., Design and fabrication of a chemically-stable proton conductor bilayer electrolyte for intermediate temperature solid oxide fuel cells (IT-SOFCs). *Energy & Environmental Science* **2008,** *1* (3), 355-359.
27. Zuo, C.; Zha, S.; Liu, M.; Hatano, M.; Uchiyama, M., Ba (Zr0. 1Ce0. 7Y0. 2) O3–δ as an electrolyte for low-temperature solid-oxide fuel cells. *Advanced Materials* **2006,** *18* (24), 3318-3320.
28. Li, W.; Shi, Y.; Luo, Y.; Wang, Y.; Cai, N., Carbon deposition on patterned nickel/yttria stabilized zirconia electrodes for solid oxide fuel cell/solid oxide electrolysis cell modes. *Journal of Power Sources* **2015,** *276*, 26-31.
29. Chen, T.; Wang, W. G.; Miao, H.; Li, T.; Xu, C., Evaluation of carbon deposition behavior on the nickel/yttrium-stabilized zirconia anode-supported fuel cell fueled with simulated syngas. *Journal of Power Sources* **2011,** *196* (5), 2461-2468.
30. Nikolla, E.; Schwank, J.; Linic, S., Direct electrochemical oxidation of hydrocarbon fuels on SOFCs: improved carbon tolerance of Ni alloy anodes. *Journal of the Electrochemical Society* **2009,** *156* (11), B1312.

Supplementary Information

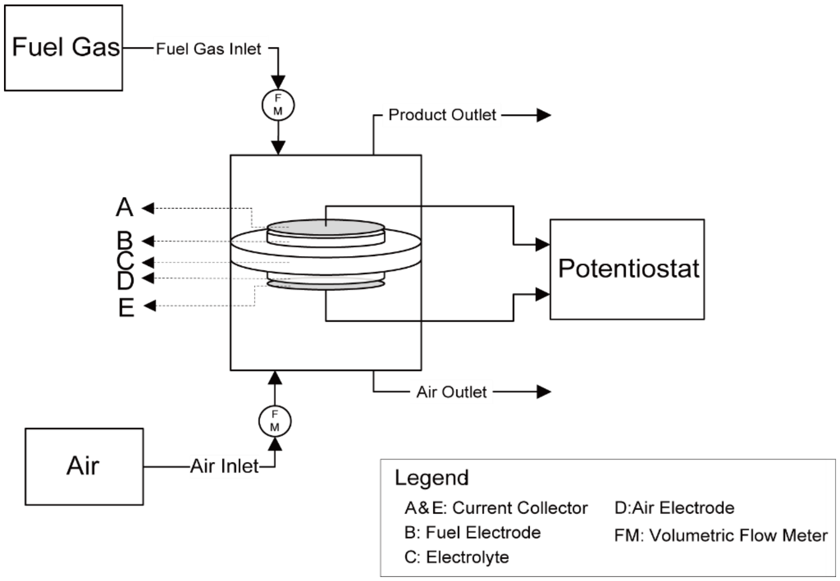

Figure S1. Schematic Diagram for electrochemical performance test setup.

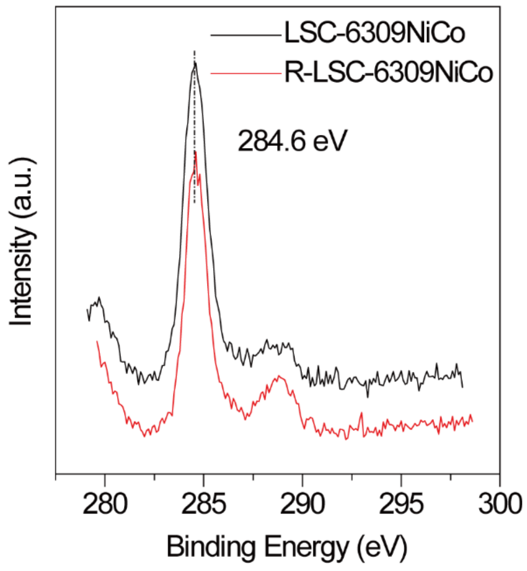

Figure S2. C1s XPS spectra of LSC-6309NiCo and R-LSC-6309NiCo.

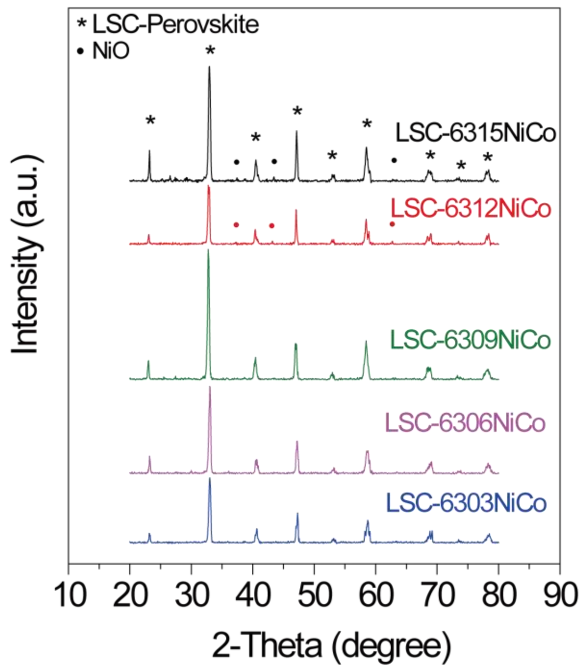

Figure S3. Crystalline structures of LSC based perovskite with different bimetallic doping ratios. The LSC-6315NiCo and LSC-6312NiCo perovskite oxides demonstrate the diffractions belonging to impurity of nickel oxide phase (reference code 01-089-7131) at 2θ equal to 37º, 43º and 63º, indicating that the saturation concentration of B-site dopants is 9 mol%.

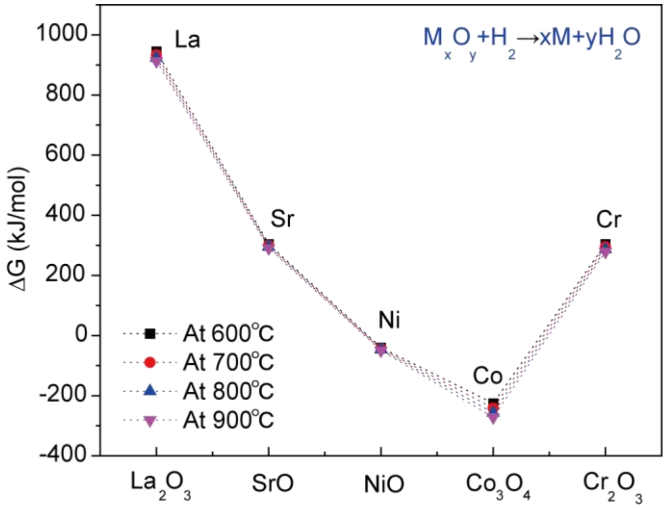

Figure S4. The theoretical value of Gibbs free energy for reduction of each element oxide in LSC-6309NiCo perovskite at 600-800 °C.

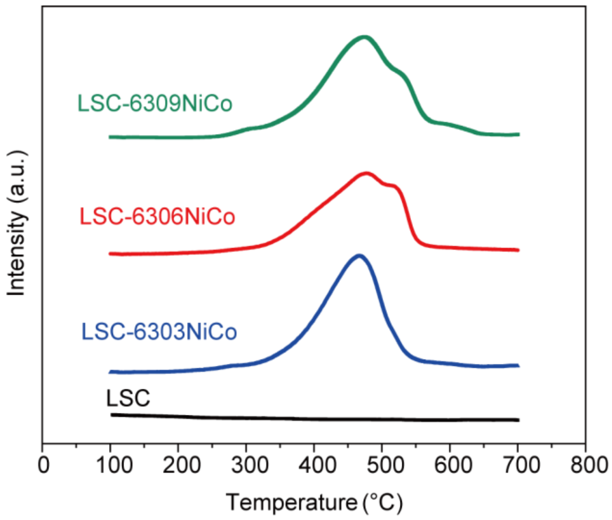

Figure S5. H_2-TPR curves of A-site deficient perovskite with different Ni/Co dopant concentrations.

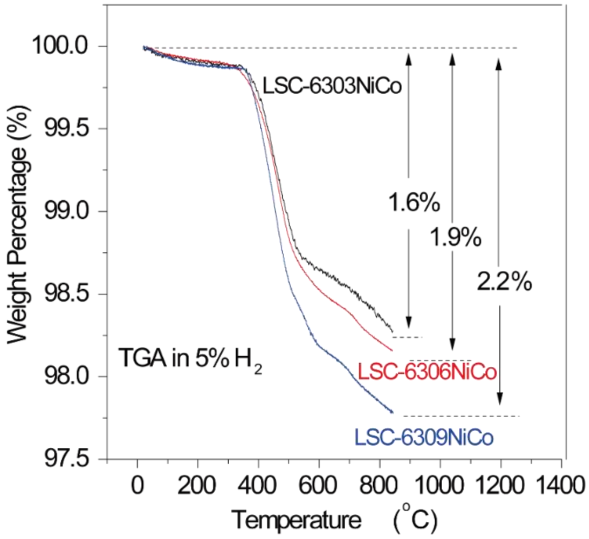

Figure S6. TGA curves of LSC 6303NiCo, LSC-6306NiCo and LSC-6309NiCo in 5% H_2/N_2. The weight losses of LSC-6303NiCo, LSC-6306NiCo, and LSC-6309NiCo reaches 1.6%, 1.9% and 2.2%, respectively.

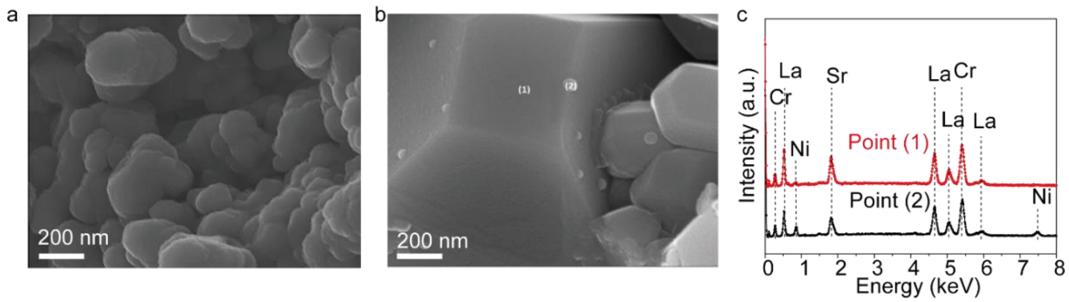

Figure S7. SEM images of a) fresh LSC-6309Ni microstructure, b) reduced sample R-LSC-6309Ni, and c) the correlated EDS point scanning results. The EDS analysis suggests its pure metallic Ni nature.

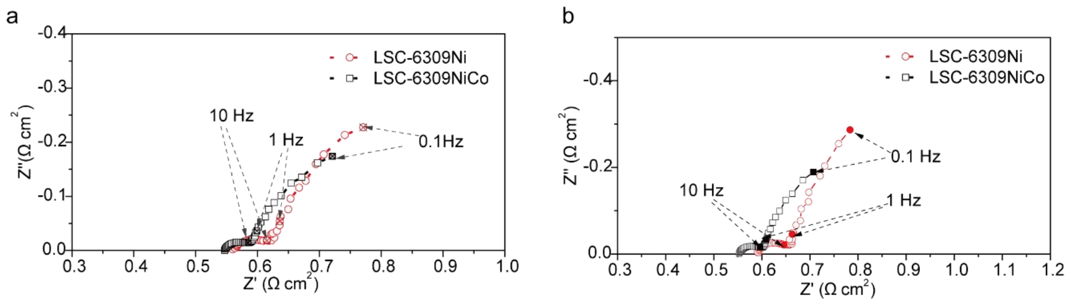

Figure S8. EIS curves of LSC-6309NiCo and LSC-6309Ni at 850°C: a) in pure H_2 gas and b) in syngas.

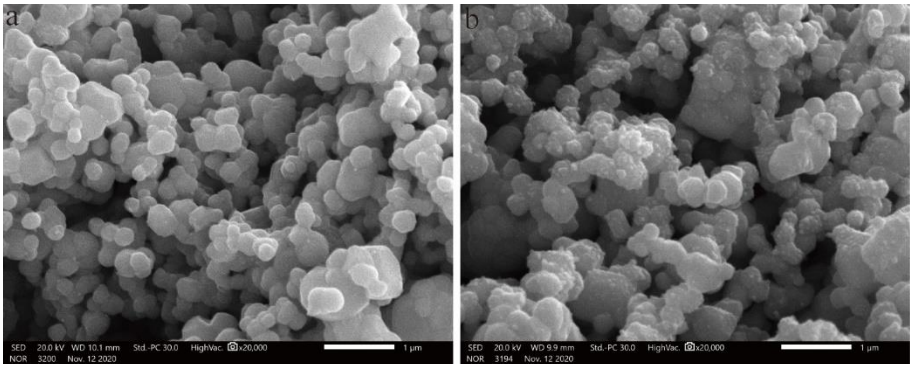

Figure S9. **SEM images of a) LSC-6309NiCo and b) LSC-6309Ni cathodes after short-term tests in 70% CO_2/30% CO at 850 °C for 30 min at constant voltages of 0.1, 0.3 and 0.5 V (vs. OCV), respectively.**

Table S1. The mole ratio of different O species based on XPS analysis.

	H_2O/CO_3^{2-}	O_2/OH^-	O_2^{2-}/O^-	M-O
LSC-6309NiCo	5.01%	38.13%	9.39%	47.47%
R-LSC-6309NiCo	1.94%	38.58%	21.50%	37.98%
